# Staying local or going back: A study on international university graduates’ mobility

**DOI:** 10.1371/journal.pone.0268821

**Published:** 2022-08-09

**Authors:** Tai Ming Wut, Jing (Bill) Xu, Carmen Ka-man Sum

**Affiliations:** College of Professional and Continuing Education, The Hong Kong Polytechnic University, Kowloon, Hong Kong; Northwestern University, UNITED STATES

## Abstract

Upon graduation, international students studying abroad often have to decide whether to stay local or return home. We investigate university graduates’ mobility from the perspective of social capital theory. An empirical study of mainland Chinese university students’ intention to remain in Hong Kong upon completion of their university studies was conducted. An online survey was used to recruit the participants. In total, 155 valid questionnaires were received, with a response rate of 40.8%. Structural capital and relational capital were found to be the important factors affecting mainland Chinese students’ intention to stay in a linear manner. Cognitive capital self-moderated the relationship between cognitive capital and intention to stay. Cognitive capital affected the intention to stay in a non-linear way. We make a unique contribution to the field by showing that 1) the effect of cognitive capital is quadratic rather than linear; 2) relational capital is more important to female students than to male students; and 3) structural capital is more important to senior year university degree students than to junior year degree students. The theoretical and managerial implications of these findings are discussed.

## Introduction

International students studying abroad must often decide whether to stay local or return home upon graduation. Global mobility refers to when individuals relocate from one country/place to another [1]. Studies have focused on the economic factors affecting the decision to move, such as employment opportunities and the cost of living in a host destination [2]. Research on how social networks affect international students’ decisions is lacking. During the COVID-19 pandemic, international students studying away from home are more fearful than home students [3]. According to social capital theory, a social network is an important resource in addition to economic resources. Social networks in the host destination could provide international students with support [4]. Therefore, social capital theory underpins the analysis of associations in this study.

More and more international students come to Hong Kong every year to pursue postgraduate degrees and bachelor degrees. Most of them are mainland Chinese students who come due to Hong Kong’s proximity to the mainland and to the use of English as a teaching language. Mainland China and other international students are required to obtain student VISA in order to pursue their studies. There are eight publicly funded universities in Hong Kong, and five of them rank within the top 100 worldwide [5]. According to the latest statistics provided by the University Grants Committee (UGC) of Hong Kong, among the 101,048 students enrolled in the 2018/19 academic year, 12,322 (12%) were from mainland China [6]. Hong Kong has had a very low birth rate, one of the lowest in the world, for the past 20 years. In 2019, Hong Kong recorded a fertility rate of just 1.0, whereas the global average rate was 2.5 [7]. The Hong Kong government therefore allows mainland Chinese students to stay and work in Hong Kong after they graduate to maintain a constant supply of workers. According to the immigration policy of Hong Kong, mainland Chinese students can extend their visa for 12 months after their undergraduate and postgraduate studies. Graduates can search for jobs within these 12-month periods [8]. Once they get a job offer, they can continue to stay in Hong Kong to develop their career. We investigate international university graduates’ mobility from the perspective of social capital theory. The boundary conditions of gender and year of study are examined.

## Literature review

### International students’ mobility

More and more students have been going aboard for higher education sector [2]. International students can be an important asset for the economic growth of their host country and attract the attention of employers. Graduates are skilful and have recognised qualifications. They also have a more global outlook when compared with local students. Scholars have therefore suggested that international students should receive help to integrate into their host destinations [9].

Some mainland Chinese students choose to study in Hong Kong due to the opportunity for personal development in a cosmopolitan city [10]. Schools in mainland China are generally less student-centred, so mainland Chinese graduates of education degrees may not be able to utilise some of the innovative teaching methods they learned in Hong Kong if they return to China [11]. Students of business-related programmes have reported that their professional competence is more valued in Hong Kong, as the working environment in mainland China places more emphasis on social networks [12].

Mainland Chinese students commonly regard Hong Kong as a transition point or stepping stone rather than their final destination. They utilise their educational experiences to realise their transnational aspirations [12]. A researcher interviewed 23 mainland students. All except one of the participants did not consider Hong Kong a place for permanent settlement, but all of them wanted to acquire maximum cultural and social capital for their life and career pursuits in Hong Kong and elsewhere upon graduation, and half of them wanted to remain in Hong Kong for a few years so that they could become permanent residents and then return to mainland China or go elsewhere. The Hong Kong government might be disappointed by this reality, because it expects these students to settle down and contribute to the local economy in return for the valuable educational and career opportunities that they receive [13].

Given the promising career developments in mainland cities due to China’s rapid economic development, attractive job offers have been found to be the strongest predictor of students’ decisions to return, followed by family ties [11]. In 2008, the central government of China introduced A Thousand Talent Plans’ to attract science and technology professionals who studied abroad to work in China by offering leadership, professional or technical positions in institutions. Benefits, including housing, medical care, comprehensive insurance packages, job opportunities for spouses and children’s admission to high-ranking schools were provided [14].

In a study of Master’s degree students from mainland China who majored in education at the Chinese University of Hong Kong, it was found that only 19% reported being ‘likely’ or ‘very likely’ to work in Hong Kong. Some decided to return to their homeland because they perceived difficulties securing stable opportunities either within or outside their profession in Hong Kong [15]. Research examining mainland Chinese students’ intention to stay in Hong Kong after the social unrest and COVID-19 outbreak is lacking. However, it is an important area of study, as most mainland Chinese students in Hong Kong (over 80%) have been using online modes of study since the beginning of year 2020. They might feel lonely and lack support from local institutions.

### Social capital theory

An original conceptualisation of social capital applies to the individual level. Social capital is a kind of personal property [16]. For example, a person might transfer his or her social connections to an advantage or a benefit, such as when a friend gives tips on a job opportunity at his or her company. In contrast, scholars positioned social capital at the group level [17, 18]. An organisation builds trust with its stakeholders to improve the efficiency of a society [19]. Thus, social capital theory provides a conceptual framework of social networks that work at the individual level, organisational level and community level [20]. It has previously been applied to the context of overseas students [4].

Social capital is ‘the sum of the actual and potential resources embedded within and derived from the network of relationships possessed by an individual or social unit’ [21, p.243]. In short, social capital can be regarded as all of the resources originating from a person’s network. We propose that mainland Chinese students who have earned higher social capital during their studies in Hong Kong may have stronger intentions to stay in Hong Kong after graduation.

Social capital is composed of ‘structural capital, relational capital and cognitive capital’ [22, p.722]. Structural capital arises from connections among people. Relational capital is the capital obtained from identification with a group, and it is determined by the quality and reliability of the connection. Cognitive capital is ‘the shared interpretations and collective visions within a group’ [22, p.722]. People within a group have similar values and goals. They share information each other.

#### Structural capital

Mainland Chinese students possess more connections than other Hong Kong students who maintain local connections only [23]. Mainland Chinese students have connections in their home towns and are able to establish new social ties in their host destinations. Despite many mainland Chinese students in Hong Kong reporting a belief that getting a part-time job or internship during their overseas studies would help them integrate better into local society, they commonly encounter difficulties securing such positions due to language and cultural differences [10]. The working language in small and medium enterprises in Hong Kong is usually Cantonese, although documents are bilingual (with English as the second language). This context provides a useful population for studying the career decisions of overseas students in Hong Kong.

In particular, mainland Chinese students’ time at university in Hong Kong is not only for pursuing academic knowledge but also for establishing networks. Structural social capital includes associations and institutions that establish connections between mainland Chinese students and people in Hong Kong. Such connections are likely to increase the likelihood of such students remaining in Hong Kong. In other words, mainland students in Hong Kong may have higher mobility with reference to some other students remaining in home town. Thus, we propose the following hypothesis:

Hypothesis 1: mainland Chinese students with higher structural capital have stronger intention to stay in Hong Kong (higher mobility).

#### Relational capital

Some mainland students may get referrals from local friends when searching for jobs (relational capital), which may also increase their likelihood of remaining in Hong Kong. In other words, these students may have higher mobility than other students based on their home town. Thus, we propose the following hypothesis:

Hypothesis 2: mainland Chinese students with higher relational capital have stronger intention to stay in Hong Kong (higher mobility).

#### Cognitive capital

When mainland Chinese students establish friendships with their classmates or other local people via internship or part-time jobs, they obtain cognitive capital as a result of their more in-depth interactions with these local people. This may give them more confidence to stay in Hong Kong to search for a job. Thus, we propose the following hypothesis:

Hypothesis 3: mainland Chinese students with higher cognitive capital have stronger intention to stay in Hong Kong (higher mobility).

Cognitive capital may have a larger effect on intention to stay because it is associated with higher levels of motivation [20]. It is possible to check for the presence of non-linear associations. Thus, we propose the following hypothesis:

Hypothesis 4: Cognitive capital has a quadratic effect on mainland Chinese students’ intention to stay in Hong Kong (higher mobility, non-linear relationship).

### Relationships among structural capital, relational capital and cognitive capital

Structural capital, relational capital and cognitive capital are interrelated [24]. It was found that social interaction can improve relationships and shared understanding. It seems that once a relationship has been established, the chances of shared behaviour increase [22]. Thus, we propose the following hypotheses:

Hypothesis 5: Structural capital is positively associated with Relational capital.
Hypothesis 6: Structural capital is positively associated with Cognitive capital.
Hypothesis 7: Relational capital is positively associated with Cognitive capital.

**Fig 1:**
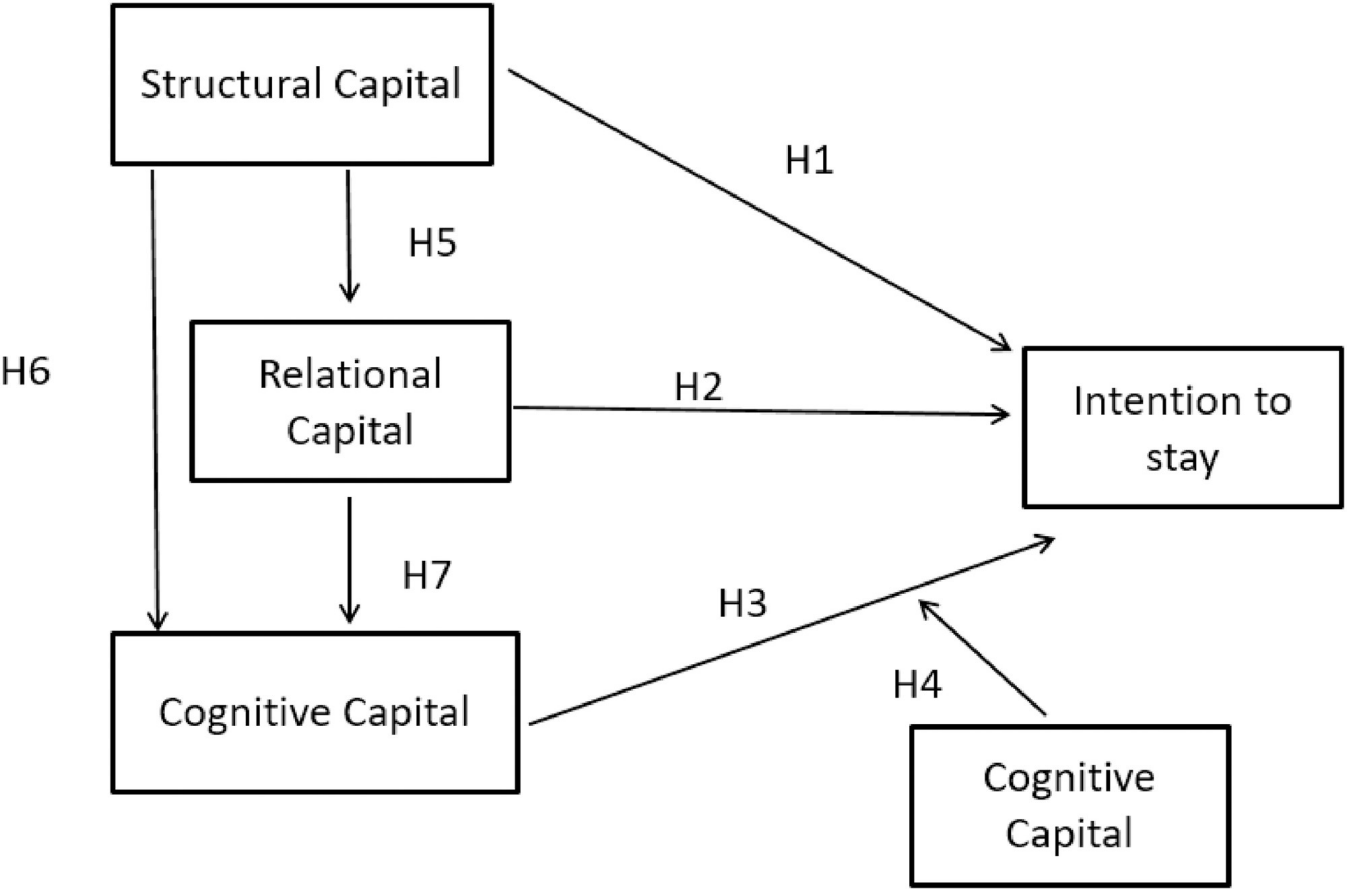
Research model.

Our research model is presented as Fig 1.

### Education level and gender

Female students often want to stay in close contact with their family members and friends from their home town, and they tend to return to their home town and work there. Only a few studies have examined the effect of gender differences on international students’ mobility [25–28].

Associate degree students usually concentrate on their studies and do not care too much about building up a network in their host destination because they still have some years before their graduation. Final year students generally care both about their academic results (which are relevant for further studies) and their local network (which relates to their intention to stay) [19]. Scholars focused on year of graduation as a moderating factor [9]. There is a research gap on the role of education level. Education level and gender are the proposed boundary conditions in our research model.

## Methodology and research design

All questions were derived from established measurements with high validity and reliability. All measurements were measured using 7-point Likert scales (1 = *strongly disagree*, 3 = *neutral*, 7 = *strongly agree*). The questionnaire contained standardised questions (Table 1). The structural, relational, cognitive capital and intention to stay constructs came from established scales [22, 29, 30]. The questionnaire started with a screening question to exclude respondents outside the target group. The screening question asked the respondents whether they had a permanent residential address in mainland China. If the answer was positive, the respondents were allowed to continue the survey. Ethical approval was obtained from the Research Committee of the College of Professional and Continuing Education, Hong Kong Polytechnic University, in January 2021.

**Table 1:** Questionnaire items.

Construct	Items	Sources
Structural capital	1. I maintain close social relationships with people in Hong Kong.	Modified from [22, 31]
	2. I know people in Hong Kong on a personal level.	
	3. I spend some time interacting with Hong Kong people.	
	4. I have frequent communication with some Hong Kong people in the virtual community.	
Relational capital	1. People in Hong Kong will do their best to help me.	Modified from [22]
	2. People in Hong Kong are reliable.	
	3. People in Hong Kong are faithful to me.	
Cognitive capital	1. People in Hong Kong exchange ideas with me.	Modified from [22]
	2. People in Hong Kong interact with me.	
	3. People in Hong Kong think that sharing information with me is pleasant.	
Intention to stay	1. I plan to stay in Hong Kong.	[32]
	2. When I have working opportunities, I will stay in Hong Kong.	

The survey was available in English and Chinese, and back translation was used to ascertain face validity. A pilot test was carried out in January 2021 before a larger-scale survey in February 2021. The wordings of three questions were modified to improve readability.

The respondents were university students from mainland China. A total of 380 emails were sent out, inviting them to take part in a survey in February 2021. Two reminders were also sent. Ultimately, 155 valid responses were collected. The overall response rate was 40.8%. A coupon of small monetary value was offered for each completed questionnaire. Early responses were compared with late responses. No significant difference in the survey results was found.

The recommended minimum sample size for the study was 137 for a minimum R-squared of 0.10 at a 5% significance level with the maximum number of arrows pointing at a construct as four. The required sample size was 65 for a minimum R-squared of 0.25 [31]. In this study, the largest number of structured paths used to examine intention to stay was four and the minimum R-squared was 0.235. Thus, the sample size requirement was fulfilled.

The sample consisted of 33.5% male and 66.5% female respondents. Most (79.4%) were 18–22 years old, 16.1% were 23–26 years old, 3.9% were 27–30 years old and 0.6% were 31 years old or above. The majority (65.8%) were business students. The others majored in social science and humanities (18.7%), information technology (6.5%), tourism/hospitality (4.5%) and education (2.6%). Most (59.4%) were top-up Bachelor’s degree students, equivalent to Year 3 and Year 4 university students in a 4-year university curriculum; 30% were higher diploma or associate degree students, equivalent to Year 1 and Year 2 undergraduate students (Table 2).

**Table 2:** Demographic data of respondents.

Category		Frequency	Percentage %
Gender	Male	52	33.5
	Female	103	66.5
Age	18–22	123	79.4
	23–26	25	16.1
	27–30	6	3.9
	31 or above	1	0.6
Education Level	Higher Diploma/Ass Degree	48	31.0
	Bachelor degree (years 3 & 4)	92	59.4
	Master degree or above	15	9.7
Field	Business	102	65.8
	Social Science & humanities	29	18.7
	Information Technology	10	6.5
	Tourism	7	4.5
	Education	4	2.6
	Science	1	0.6
	Medical	1	0.6
	Engineering	1	0.6
	Others	0	0.0
	Total	155	100

Structural equation modelling (SEM) was used to verify our proposed hypotheses and research model. SmartPLS (version 3.2.9) was used to compute the path coefficients.

SEM is one of the statistical tools most commonly used to examine the framework of a parsimonious model. A parsimonious model is a simplified framework that aims to explain an observed phenomenon. ‘It is claimed to be more powerful than other multivariate techniques including multiple regression. One dependent variable may be an independent variable in other dependence relationships’ [32, p. 650].

Reflective indicator loadings of variables must be higher than 0.708 to ensure reliability. Cronbach’s alpha and composite reliabilities are used to check the internal consistency with a recommended threshold of 0.70 [33]. The average variance extracted (AVE) of variables should be higher than 0.50 to indicate convergent validity [35]. All of the constructs in this study met these requirements. Fornell–Larcker’s criterion was used to check the discriminant validity of the constructs [34].

The path coefficients were measured via bootstrap analysis. To confirm the structural model, the coefficient of determination (*R*^2^) and the blindfolding-based cross-validated redundancy measure (*Q*^2^) with consideration of the effect size (*f*^2^) were used [35]. *Q*^*2*^ values of 0.5, 0.25 and 0 imply high, medium and low predictive relevance of the model, respectively (Hair et al., 2019). *f*^2^ values of 0.35, 0.15 and 0.02 represent high, medium and low effect sizes, respectively [35].

## Findings

### Assessment of measurement model fit

Table 3 shows the reflective measurement model assessment of the four constructs. The indicator loadings were all higher than the recommended threshold of 0.708. Loading results ranging from 0.788 to 0.955 were obtained for the reflective measurement models, showing that more than half of the indicators’ variances were explained and provided good item reliability. The Cronbach’s alphas (0.870 to 0.928) and composite reliabilities (0.938 to 0.954) of all of the variables exceeded the benchmark, indicating a satisfactory result. The AVE measures of all of the variables ranged from 0.790 to 0.894, exceeding the recommended threshold of 0.50. The results indicate that the constructs explained almost 80% of the variance of the related items. They also indicate sufficient convergent validity. Finally, Fornell–Larcker’s criterion was fulfilled because the square root of the AVE of each construct was greater than the construct’s highest correlation with any other construct (Table 4). This finding implies that all of the constructs were reliable and valid. Subsequently, the structural model was assessed.

**Table 3:** Measurement model assessment.

Construct	Item	Loading	Cronbach’s alpha	Composite Reliability	AVE
Structural Capital	sc1	0.902	0.910	0.938	0.790
	sc2	0.788			
	sc3	0.917			
	sc4	0.941			
Relational Capital	rc1	0.928	0.928	0.954	0.874
	rc2	0.944			
	rc3	0.933			
Cognitive Capital	Cc1	0.924	0.933	0.957	0.881
	Cc2	0.957			
	Cc3	0.935			
Intention to stay	Stay1	0.955	0.883	0.944	0.894
	Stay2	0.936			

**Table 4:** Assessing discriminant validity.

Construct	Mean	SD	Cognitive Capital	Intention to stay	Relational Capital	Structural Capital
Cognitive Capital	4.79	1.47	**0.939**			
Intention to stay	4.60	1.46	0.282	**0.946**		
Relational Capital	5.12	1.24	0.856	0.338	**0.935**	
Structural Capital	4.80	1.54	0.775	0.385	0.682	**0.889**

Note: Bold means that the square root of the AVE of each construct is greater than the construct’s highest correlation with any other construct.

### Assessment of structural model

The structural model was supported with satisfactory results. The *R*^*2*^ values of Cognitive capital, Relational capital and intention to stay were 0.801, 0.465 and 0.235, Thus, 23.5% to 80.1% of the variances were explained, indicating weak to strong in explanation. The *Q*^*2*^ values ranged from 0.165 to 0.664, indicating medium to high predictive relevance of the path model. A similar result was obtained using PLSpredict procedure. The *Q*^*2*^ values ranged from 0.108 to 0.554 using holdout samples. Finally, all *f*^2^ effect sizes of predictor construct ranged from 0.037 to 1.000 (except unsupported hypotheses), indicating small to large effect sizes on intention to stay.

**Fig 2:**
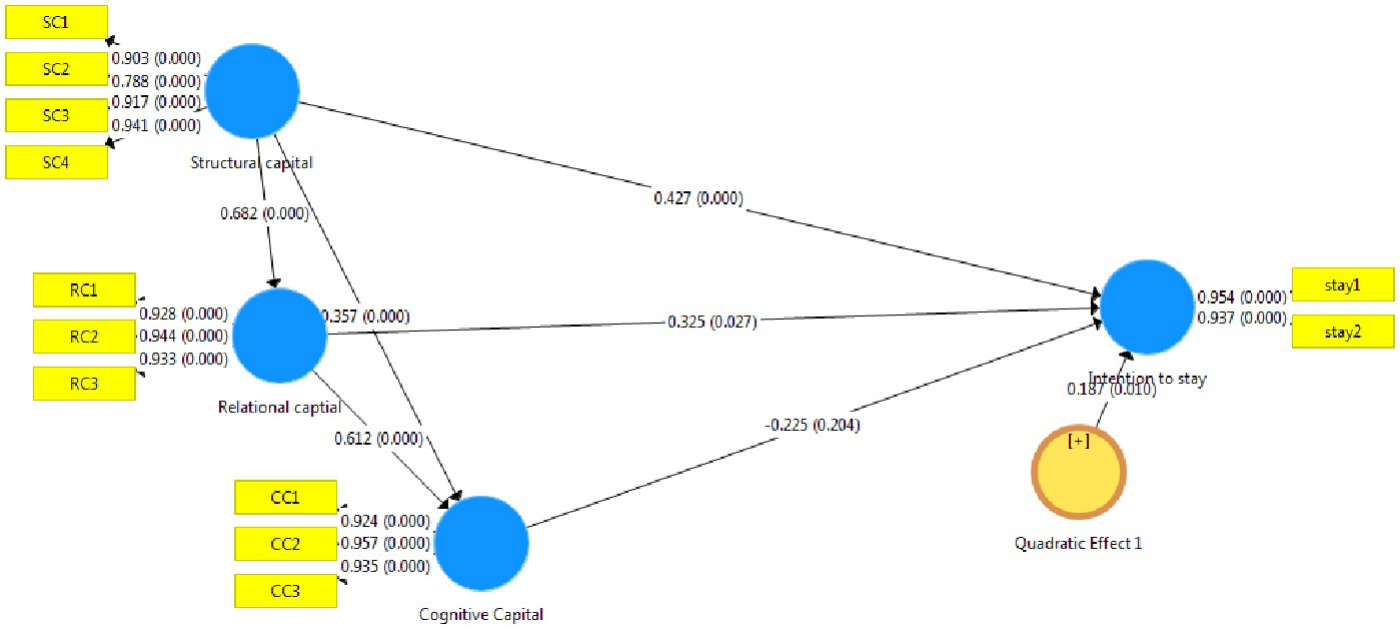
Partial least squares SEM model (source: Authors).

The path coefficients and *t*-values were evaluated by conducting bootstrap analysis with 5,000 subsamples for the 155 cases (Fig 2).

**Fig 3:**
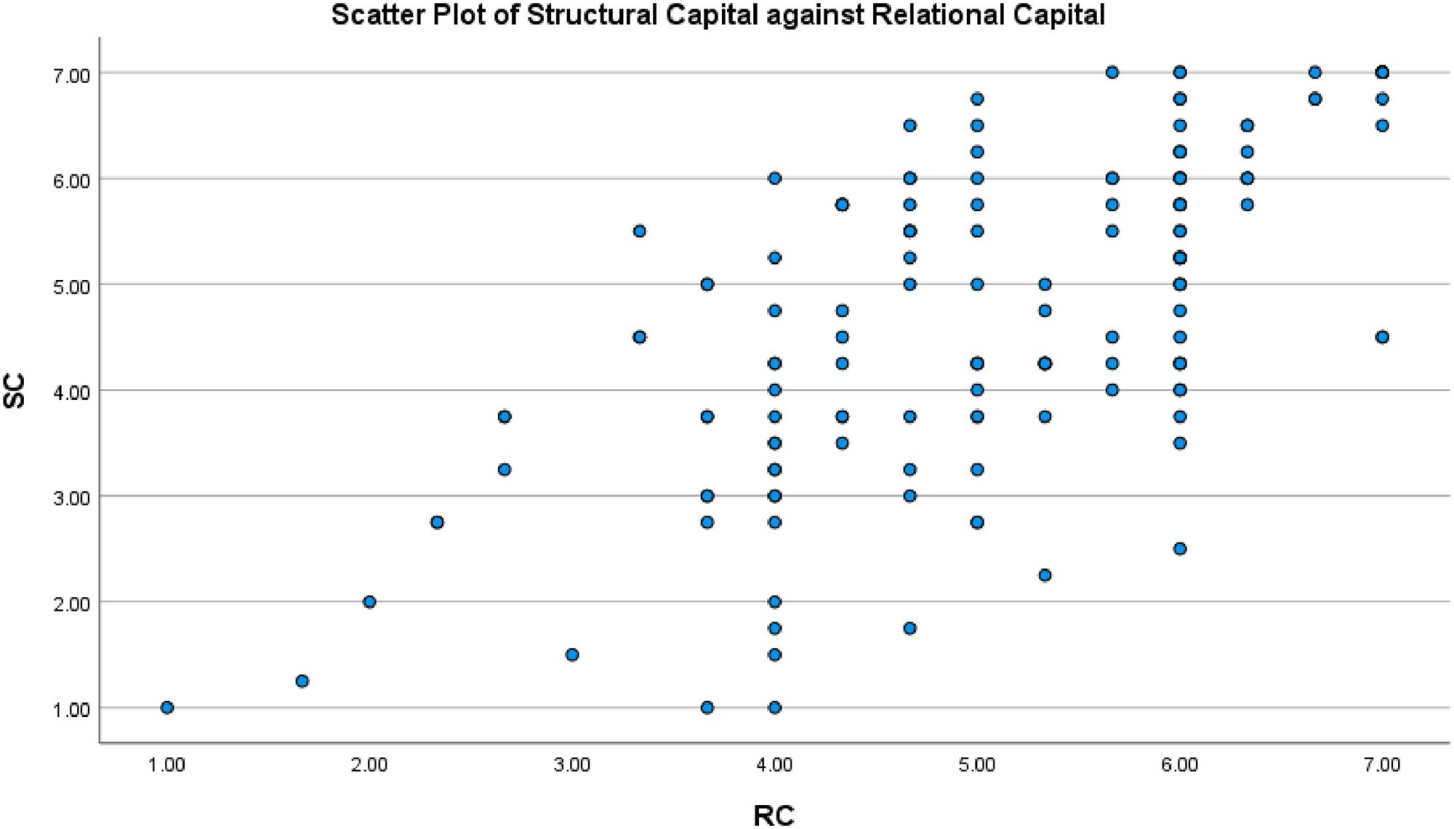
Scatter plot between the structural capital and relational capital.

**Fig 4:**
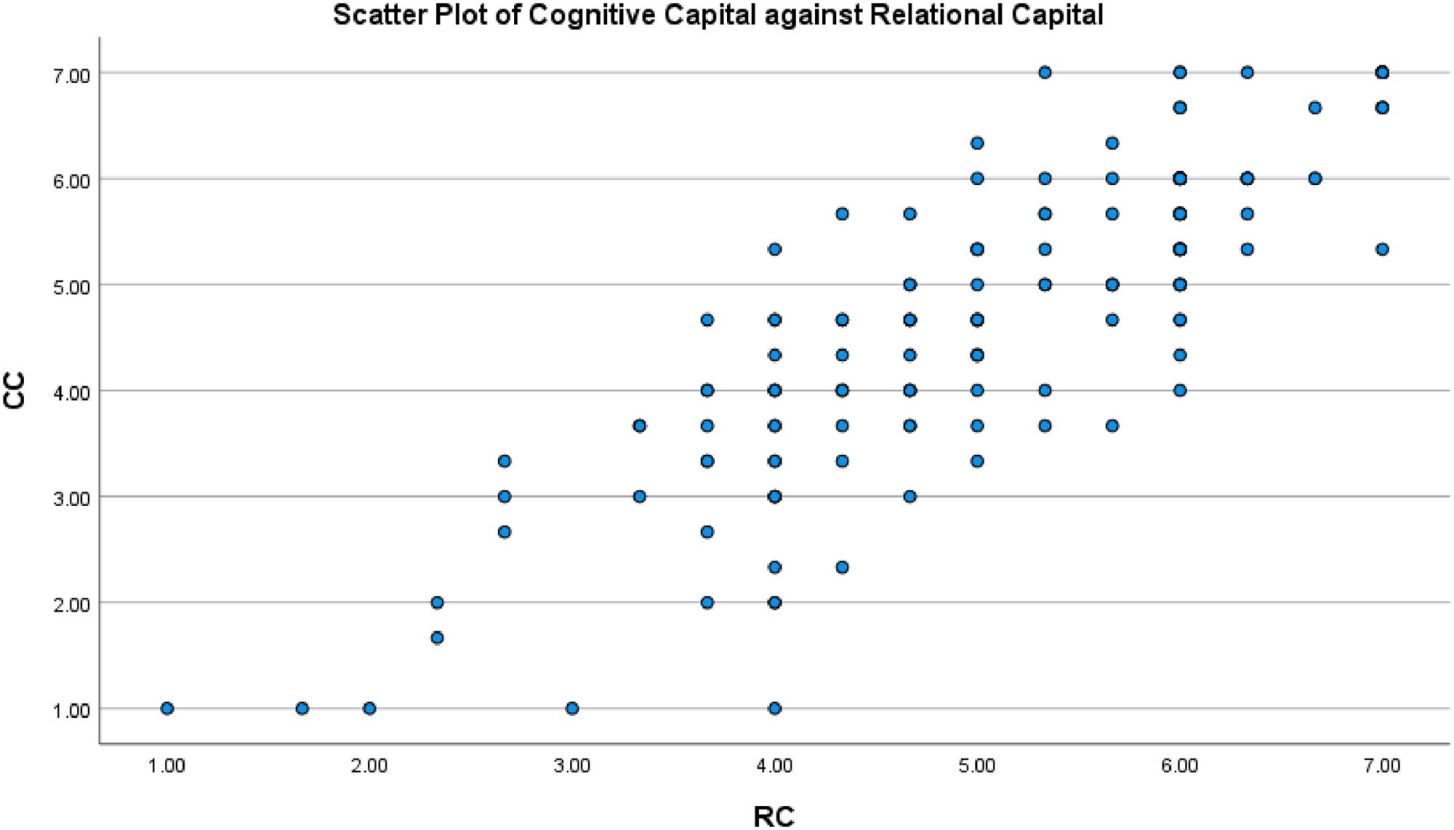
Scatter plot between the cognitive capital and relational capital.

**Fig 5:**
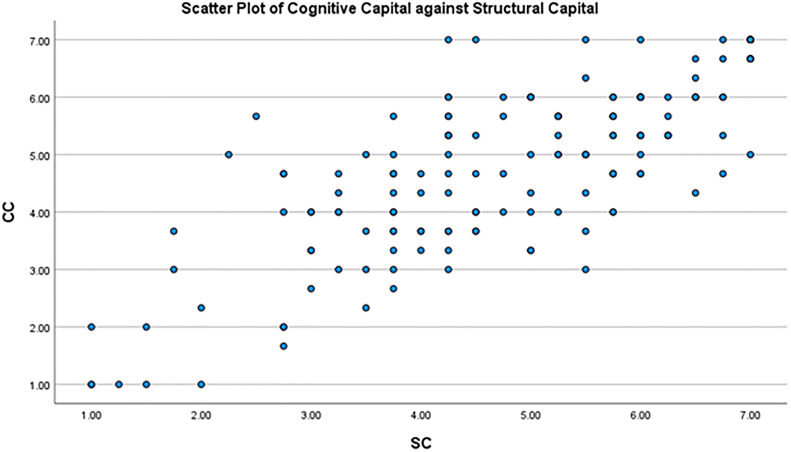
Scatter plot between the cognitive capital and structural capital.

Scatter plots of structural capital, relational capital and cognitive capital were prepared, showing an approximately linear relationship among variables (Figs 3–5).

### Results of hypotheses testing and path analysis

Intention to stay in host place was the outcome of the research model. Structural capital, relational capital and cognitive capital were the antecedents. The following hypotheses test results addressed the direct relationships among these constructs (Table 5).

**Table 5:** Results of hypothesis testing.

Hypothesis	Path	Path Coefficient	t-value	p-Value	Result
Hypothesis 1	Structural capital → Intention to stay	0.427	3.632	0.0004***	Supported
Hypothesis 2	Relational capital → Intention to stay	0.325	2.207	0.027*	Supported
Hypothesis 3	Cognitive capital → Intention to stay	-0.225	1.271	0.204	Unsupported
Hypothesis 4	Cognitive Capital → Intention to stay (Quadratic effect)	0.187	2.591	0.010*	Supported
Hypothesis 5	Structural capital → Relational capital	0.682	15.512	0.0004***	Supported
Hypothesis 6	Structural capital →Cognitive capital	0.340	6.071	0.0004***	Supported
Hypothesis 7	Relational capital →Cognitive capital	0.614	12.062	0.0004***	Supported

(Bootstrap samples = 5000, n = 155 cases).

*p < 0.05,

***p < 0.001.

As we tested several hypotheses, the Benjamini and Hochberg method was adopted to control the false discovery rate. For Hypothesis 2, 0.027 < 0.05 x 6/7. For Hypothesis 4, 0.01 < 0.05 x 5/7. Thus, the requirements were fulfilled for both hypotheses.

Hypothesis 1 proposes a relationship between structural capital and intention to stay. As shown in Table 5, this hypothesis was supported (*p* = 0.002), indicating that structural capital positively influences mainland Chinese students’ intention to stay in Hong Kong upon graduation.

Hypothesis 2 proposes a positive association between relational capital and intention to stay. As shown in Table 5, a positive and significant relationship was confirmed (*p* = 0.054), indicating that relational capital drives mainland Chinese students’ intention to stay in Hong Kong.

Hypothesis 3 proposes a positive relationship between cognitive capital and intention to stay. As shown in Table 5, this hypothesis was not supported (*p* = 0.502), indicating that cognitive capital does not influence mainland Chinese students’ intention to stay in Hong Kong.

Hypothesis 4 proposes a non-linear association between cognitive capital and intention to stay. As shown in Table 5, a positive and significant relationship was confirmed (*p* = 0.005), indicating that cognitive capital drives mainland Chinese students’ intention to stay in Hong Kong in a quadratic manner.

Hypothesis 5 proposes a positive relationship between structural capital and relational capital. As shown in Table 5, this hypothesis was supported (*p* < 0.001), indicating that structural capital positively influences relational capital.

Hypothesis 6 proposes a positive association between structural capital and cognitive capital. As shown in Table 5, a positive and significant relationship was confirmed (*p* < 0.001), indicating that structural capital drives mainland Chinese students’ cognitive capital in Hong Kong.

Hypothesis 7 proposes a positive relationship between relational capital and cognitive capital. As shown in Table 5, this hypothesis was supported (*p* < 0.001), indicating that relational capital is positively associated with cognitive capital.

### Multi-group analysis

Educational-level differences were observed among the results of the hypotheses (Table 6). In the sample, 48 respondents (31%) were studying associate degrees and 92 (59.4%) were studying senior-year Bachelor’s degrees (equivalent to Year 3 and Year 4). The structural capital of the Year 3 and Year 4 degree students (β = 0.670, *t* = 3.750, *p* < 0.001) was strongly associated with intention to stay. No similar significant findings were observed for the associate degree students. Similarly, the cognitive capital of the senior year students was strongly associated with intention to stay in a non-linear manner. No similar findings were observed for the associate degree students. Furthermore, the relational capital of the associate degree students (β = 0.624, *t* = 2.107, *p* = 0.035) was strongly associated with intention to stay. No similar findings were observed for the senior year students. Regarding Hypothesis 7, the associate degree students demonstrated a stronger relationship between relational capital and cognitive capital than the senior year degree students.

**Table 6:** Multi-group analysis (education level).

Hypothesis	Associate Degree			Degree		
	Beta	t-value	p-value	Beta	t-value	p-value
H1	0.076	0.425	0.671	0.670	3.750	0.0004***
H2	0.624	2.107	0.035*	0.140	0.675	0.5 (ns)
H3	-0.195	0.611	0.542 (ns)	-0.274	1.041	0.298 (ns)
H4	0.043	0.318	0.750 (ns)	0.286	3.136	0.002**
H5	0.585	6.646	0.000***	0.727	15.535	0.0004***
H6	0.273	3.063	0.002**	0.394	5.261	0.0004***
H7	0.701	8.697	0.000***	0.286	8.116	0.0004***

**p < 0.01,

***p < 0.001.

The hypothesis tests indicated some gender differences (Table 7). The relational capital of the female respondents was associated with intention to stay (β = 0.367, *t* = 1.893, *p* = 0.058). No similar findings were observed for the male respondents, indicating that there is a significantly stronger relationship between structural capital and relational capital for female students than for male students.

**Table 7:** Multi-group analysis (gender).

Hypothesis	Male			Female		
	Beta	t-value	p-value	Beta	t-value	p-value
H1	0.419	2.419	0.016*	0.397	2.455	0.014*
H2	0.253	1.159	0.246 (ns)	0.367	1.893	0.058+
H3	-0.167	0.601	0.548 (ns)	-0.275	1.261	0.207 (ns)
H4	0.043	2.568	0.010*	0.286	2.378	0.017*
H5	0.494	4.666	0.000***	0.744	17.767	0.0004***
H6	0.321	3.757	0.000***	0.363	4.801	0.0004***
H7	0.671	8.435	0.000***	0.593	8.370	0.0004***

^+^ p< 0.1,

*p < 0.05,

***p < 0.001.

## Discussion

Among the three dimensions of social capital, the structural and relational capital of students from mainland China was linearly associated with their intention to stay in Hong Kong (Hypotheses 1 and 2). Cognitive capital was non-linearly associated with intention to stay (Hypothesis 4). This is likely to be because shared understanding requires a higher-level interaction and thus may have a greater impact on students’ intention to stay. Students’ intention to stay can be expressed as follows:

Student intention to stay in host destination = 0.427 (structural capital) + 0.325 (relational capital) + 0.187 (cognitive capital)^2^

Hypotheses 5, 6 and 7 were also supported, in agreement with the literature [22]. This shows that structural capital is an important antecedent of cognitive capital and relational capital. More importantly, cognitive capital indicates a shared meaning between mainland Chinese students and Hong Kong students, which suggests a high level of interaction. Relational capital serves as a mediator between structural capital and cognitive capital.

The results suggest that female students’ relationship building is more effective. It has been found that female students speak up more in online environments than in face-to-face classes [35]. This may be because the Internet provides a comfort zone for female students [36]. Online teaching was the dominant mode of delivery for the course material in this study. This may explain why relational capital affected the intention to stay of the female students but not of the male students.

Final year and Year 3 students need more personal networks (structural capital) to facilitate their job search. For associate degree (first- or second-year) students, such networks might not be as important, as they have 3 or 4 more years to get to know more locals. Associate degree students typically aim to upgrade to degree students to gain a more competitive edge in the job market. A similar argument can be made for the quadratic effect of cognitive capital. Senior year students also are encouraged joining a group with similar vision.

**Fig 6:**
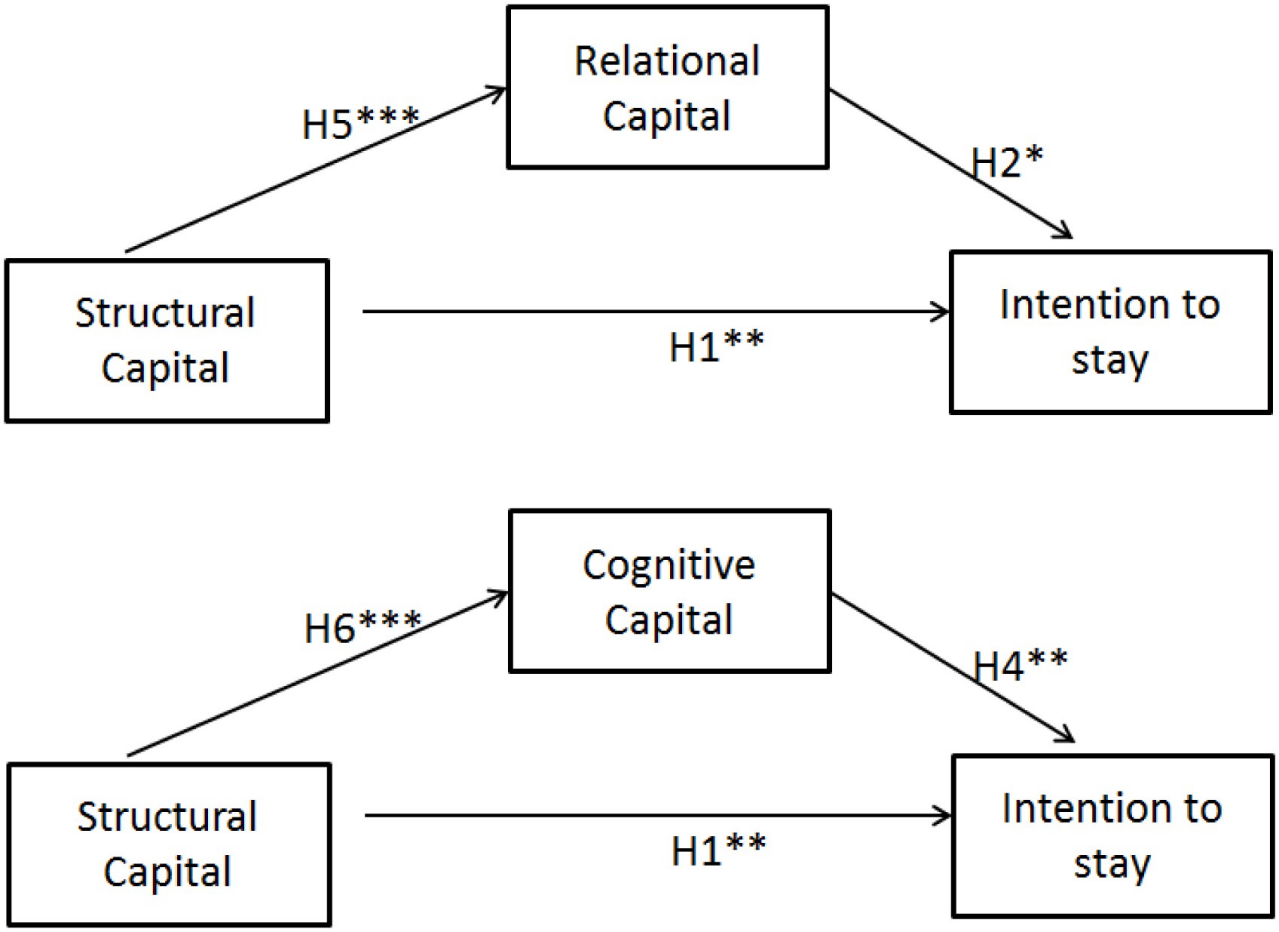
Partial mediation effects.

In this study, the effect of structural capital on intention to stay was mediated by both relational capital and cognitive capital. Hypothesis 5, which proposes that structural capital and relational capital are associated, was supported. Relational capital was also associated with intention to stay (Hypothesis 2), demonstrating a partial mediation effect (Fig 3). Hypothesis 6, which proposes a positive association between structural capital and cognitive capital, was also supported. Cognitive capital was associated with intention to stay (Hypothesis 4), demonstrating a partial mediation effect (Fig 6).

## Theoretical and managerial implications

Our results show that compared to relational capital and cognitive capital, structural capital is the most important factor affecting mainland Chinese students’ intention to stay in Hong Kong. It is not surprising that a strong personal network can significantly help students’ career, as most of the jobs in Hong Kong are related to the commercial field. An informal network provides a good foundation for success. In addition to the direct effect of structural capital, structural capital was also found to affect the students’ intention to stay in the host destination through indirect effects of relational capital and cognitive capital.

Unlike previous studies, cognitive capital had a quadratic effect on the students’ intention to stay, implying that a deeper understanding between visiting students and local students plays an important role.

Policy makers who want to attract more students to stay in host destinations should help such students expand their personal networks. This could be achieved by organising activities to encourage students to make local friends. As well as expanding students’ networks, in-depth exchanges are important. One-to-one mentorship could provide intensive exchanges, and teachers or graduates could act as mentors to help cross-border university students adapt to the new environment of their host destination.

## Conclusion

We make a unique contribution to the field by using social capital theory to examine why international students choose to study outside their home town and remain in their host destination. Structural capital and relational capital affected intention to stay in a linear manner, and cognitive capital affected it in a quadratic manner. Relational capital and cognitive capital mediated the relationship between structural capital and intention to stay. Relational capital appears to be more important to female students than to male students, and structural capital is more important to final year or Year 3 students than to associate degree (Year 1 or Year 2) students.

In particular, mainland Chinese students in Hong Kong were investigated, with Hong Kong serving as the host destination in this study. Mainland China and Hong Kong share some common characteristics of language and culture. Future studies could use the United States or the United Kingdom as the host countries for comparison purposes. We focused on how social ties affect students’ intention to stay. However, other factors that might affect their intention to stay were not examined.

Lastly, we did not consider the students’ backgrounds or work experiences in this study. Future research could explore the possible influences of age, character, mindset, self-motivation and local internship experiences on intention to stay in a host destination for work. More students majoring in subjects other than business could also be included in a future study to examine other possible boundary conditions.

## Supporting information

S1 Questionnaire (DOCX)

S1 File (CSV)
