# Supplementary material for: Staying local or going back: A study on international university graduates’ mobility
Source: PLoS One. 2022 Aug 9;17(8):e0268821. doi: 10.1371/journal.pone.0268821 (PMC9362924; doi:10.1371/journal.pone.0268821)
Supplement: S1 Questionnaire — (DOCX) [file pone.0268821.s001.docx]

Dear student,

We are conducting an empirical survey looking at Hong Kong University Students. Your participation will be most useful to our research project.

Please kindly spare few minutes to complete the following questions. All data collected will be kept anonymous and strictly used for the academic purpose. Only the researchers or authorized person can access the data. The data will be kept for not more than seven years after the publication of research results. Participation is entirely voluntary. We highly appreciate your contributions to this research.

If you have any questions about this research project, please feel free to contact Miss Catherine Chan at 3746-0732.

Your help is very much appreciated!

Edmund Wut, Bill Xu, Carmen Sum

The Hong Kong Polytechnic University

同學，您好！

我們現正進行一項有關香港大學生的調查。您的參與將對我的研究項目極有幫助。

您需要完成一份問卷。你所回答的所有答案將會以匿名方式作研究及保密。研究報告中，將不會披露或識別受訪者的身份。這些數據只有研究人員或授權人士方可處理，並且在研究結果發表後最多只保留七年。請放心，我們絕不會將您的身份透露給任何人。參與本研究純屬自願性質，這表示您可以選擇隨時退出而不會引起任何負面影響。

如對此研究項目有任何疑問，歡迎致電3746-0732向陳瑋彤小姐查詢。

非常感謝您的協助！

屈大明、徐景、沈嘉敏

香港理工大學

a. I understand my rights and agree to participate in this survey voluntarily.

本人知悉我的權利並同意自願參加本問卷調查。☐ Yes知道 ☐ No 不知道

b. Are you a student have a permanent address in mainland China studying at or graduated from tertiary institute(s) in Hong Kong? 你是擁有內地居住地址的香港大專院校就學或畢業的學生？ ☐ Yes是 ☐ No 否

**Part I**

|  | Strongly Disagree  非常  不同意 | |  | | | | | Strongly  Agree  非常  同意 | |
| --- | --- | --- | --- | --- | --- | --- | --- | --- | --- |
|  | 1 | 2 | | 3 | 4 | 5 | 6 | | 7 |
| **Structural Capital聯繫資本** | | | | | | | | | |
| 1. I maintain close social relationships with people in Hong Kong.   我與香港人保持密切的社交關係。 |  |  | |  |  |  |  | |  |
| 1. I know people in Hong Kong on a personal level. 我在香港有相熟的人。 |  |  | |  |  |  |  | |  |
| **Relational Capital人際資本** | | | | | | | | | |
| 1. People in Hong Kong will do their best to help me. 香港人會盡力幫助我。 |  |  | |  |  |  |  | |  |
| 1. People in Hong Kong are reliable.   香港人值得信賴。 |  |  | |  |  |  |  | |  |
| 1. People in Hong Kong are faithful to me.   香港人坦誠對待我。 |  |  | |  |  |  |  | |  |
| **Cognitive Capital 認知資本** | | | | | | | | | |
| 1. People in Hong Kong exchange ideas with me. 香港人與我交流思想。 |  |  | |  |  |  |  | |  |
| 1. People in Hong Kong interact with me.   香港人與我互動。 |  |  | |  |  |  |  | |  |
| 1. People in Hong Kong think that sharing information with me is pleasant.   香港人樂於與我分享資訊。 |  |  | |  |  |  |  | |  |

**Part II: Personal Characteristics個人特點**

**Past Experience過去經歷**

1. How long did you work in Hong Kong and/or place other than mainland China before? (included both full time and part-time) 您曾在香港及/或中國內地以外的地方工作多久？ （包括全職和兼職）

☐ None 無 ☐ Less than 6 months 少於六個月 ☐ 6 months or more but less than 1 year Less than 1 year 少於一年 ☐ 1 year or more but less than 2 years 一至二年 ☐ 2 years or more but less than 3 years二至三年 ☐ 3 years or more but less than 4 years三至四年 ☐ 4 years or above 四年或以上

|  | Strongly Disagree  非常  不同意 | |  | | | | | Strongly  Agree  非常  同意 | |
| --- | --- | --- | --- | --- | --- | --- | --- | --- | --- |
|  | 1 | 2 | | 3 | 4 | 5 | 6 | | 7 |
| **Family Ties家庭關係** | | | | | | | | | |
| 1. My family members are mainly in mainland. 我的家人都在大陸。 |  |  | |  |  |  |  | |  |
| **Job availability 工作機會** | | | | | | | | | |
| 1. Hong Kong has available job positions which is related to my study here.   香港有與我修讀科目相關的職位。 |  |  | |  |  |  |  | |  |
| **Stay Intentions 逗留意向** | | | | | | | | | |
| 1. I plan to stay in Hong Kong.   我打算留在香港。 |  |  | |  |  |  |  | |  |
| 1. When I have the opportunities, I will stay in Hong Kong.   如果有機會，我會留在香港。 |  |  | |  |  |  |  | |  |

**Part III: Demographics 個人資料**

1. **Gender 性別** ☐ Male 男 ☐ Female女
2. **Age 年齡** ☐18-22 years old 18至22歲 ☐ 23-26 years old 23至26歲

☐ 27-30 years old 27至30歲 ☐ 31 years old or above 31歲或以上

1. **Education Level (attending) 教育程度(就讀)**

☐Diploma/ Associate Degree / Higher Diploma文憑/副學士/高級文憑

☐Bachelor本科 ☐Master or above碩士及以上

17**. Field of study 修讀學系** □ Accounting, business and finance會計、商業及金融

□ Humanities and social Science人文及社會科學 □Pure science純科學

□ Tourism and hospitality旅遊及款待 □Education 教育

□ Medical related studies醫療及護理

□ Logistics supply chain物流及供應鏈 □ Information technology 資訊科技

□ Engineering, surveyor and property management工程、測量及物業管理

☐ Others (please specific) 其他（請註明）______________________

***This is the end of survey and thanks for your cooperation. 問卷完畢，感謝您的協助！***
